# The Kelch-Repeat Superfamily Gene *SiNL4* Regulates the Leaf Width in Foxtail Millet

**DOI:** 10.3390/plants15121826

**Published:** 2026-06-12

**Authors:** Yuqin Zhao, Yixuan Ma, Yanyu Yang, Lejie Yang, Lu Chen, Tianguo Wang, Shiyuan Wang, Kai Zhao, Xiaorui Li, Shuqi Dong, Hongzhi Wang, Xiaoqian Chu, Jiagang Wang, Lulu Gao, Guanghui Yang

**Affiliations:** College of Agronomy, Shanxi Agricultural University, Special Orphan Crops Research Center of the Loess Plateau, MARA, Jinzhong 030800, China

**Keywords:** Kelch-repeat superfamily, leaf width, yield, foxtail millet, *SiNL4*

## Abstract

The Kelch-repeat superfamily genes played important roles in regulating plant growth and development; however, their functions in foxtail millet (*Setaria italica*) have not yet been characterized. In this study, *SiNL4*, a homolog of *ZmNL4* controlling leaf width in maize, was knocked out using the CRISPR/Cas9 technology, and two homozygous knockout lines (*ko1* and *ko2*) were obtained. Phenotypic analysis showed that compared with the wild-type Ci846, *ko1* and *ko2* exhibited reduced leaf width and decreased yield related traits (e.g., panicle weight, grain width, and 1000-grain weight). Cytological analysis showed that changes in leaf width of *ko1* and *ko2* resulted from a decrease in leaf epidermal cell width and the number of small vascular bundles (SVBs) close to the leaf edge, suggesting that *SiNL4* might regulate leaf width by influencing cell expansion and the development of SVB. Spatiotemporal expression analysis indicated that the relative expression level of *SiNL4* was high in the stem, leaf, and young panicle. Subcellular localization showed that SiNL4 was mainly localized in the mitochondria and plasma membrane. In addition, the T-DNA insertion mutant (*Atnl4*) of *AT5G18590*, the ortholog of *SiNL4* in *Arabidopsis thaliana*, exhibited similar phenotypes with reduced rosette leaf width, seed width, and 1000-seed weight. Moreover, complementary expression of *SiNL4* in *Atnl4* not only restored the phenotypes, but also significantly increased the 1000-seed weight, indicating that the function of these two genes might be conserved. Meanwhile, we found that *SiNL4* knockout caused a decrease in chlorophyll content and net photosynthetic rate (*Pn*), showing that *SiNL4* might be involved in regulating photosynthesis. In summary, this study revealed the function of *SiNL4* in regulating leaf width in foxtail millet, providing a potential gene for the genetic improvement of foxtail millet.

## 1. Introduction

Foxtail millet (*Setaria italica* L.), one of the oldest domesticated crops originating from Northern China, has long been valued for its remarkable tolerance to abiotic stresses such as drought, salinity and poor soils [1]. Foxtail millet has a small diploid genome (~500 Mb) and a short life cycle, making it an ideal model for studying C4 photosynthetic efficiency and stress biology [2,3]. In addition, its grains serve as a nutrient-rich functional food, and its straw is a high-quality forage source [4]. Despite these advantages, foxtail millet has shifted from a staple food to a minor cereal over the past decades, with a dramatic decline in cultivation area largely driven by relatively low yield potential and limited mechanized production [5]. Therefore, breeding elite cultivars with improved plant architecture and higher grain yield is a central priority for foxtail millet production.

The leaf was the main organ responsible for photosynthesis and a proper leaf width was a critical plant architecture trait for improving the efficiency of light absorption and conversion [6]. Deciphering the genetic and molecular mechanisms regulating leaf width would contribute to optimizing plant architecture and ultimately improving grain yield [7]. Leaf development is a highly coordinated process involving the precise regulation of cell division, cell expansion, and vascular patterning [8]. In recent years, a number of genes regulating leaf width have been cloned in crops. Phytohormone signaling pathways play prominent roles: auxin-related genes such as *NAL7* [9] (encoding a YUCCA family flavin monooxygenase), the auxin response factor *OsARF19*, and the auxin polar transport regulator *NAL1* [10] modulate leaf width and vein formation. Cytokinin signaling affects leaf patterning, as shown by mutants of the histidine kinase *ZmHK1* in maize [11]. Gibberellins promote cell elongation and their biosynthesis or signaling mutants, such as rice *d1* and *pla1*, exhibit altered leaf width [12,13]. Brassinosteroids regulate leaf erectness and width through conserved pathways involving *BRI1*, *BZR1*, and downstream components [14,15]. In addition to hormones, several transcription factors (e.g., the WOX family gene *OsWOX3A* [16] and the KANADI factor *SLL1* [17]) and microRNAs (e.g., *miR319* targeting *TCP* genes [18]) are critical for leaf width determination. At the cellular level, genes affecting cell wall synthesis or chloroplast development also contribute to leaf width variation [19,20]. Despite these advances, relatively few genes have been functionally characterized for leaf width regulation in foxtail millet, limiting our understanding of leaf morphogenesis in this important C4 crop.

The Kelch-repeat superfamily represents a large and diverse group of proteins characterized by a β-propeller structure formed by multiple Kelch-repeat motifs [21]. In plants, Kelch-repeat proteins participate in a wide range of biological processes, including hormone signaling [22], light perception [23], secondary metabolism [24,25], and stress responses [26], as comprehensively reviewed by Hassan et al. [27,28]. The Kelch-repeat-containing F-box (FBK) subfamily, such as ZTL and FKF1, functions as substrate recognition subunits of SCF E3 ubiquitin ligase complexes to mediate protein degradation [29]. Another subfamily, Kelch-ACBP proteins, contains C-terminal Kelch repeats and an N-terminal acyl-CoA-binding domain, and members such as *Arabidopsis* ACBP4 and ACBP5 localize to the cytosol to bind oleoyl-CoA and phospholipids [30]. Recently, a Kelch-repeat superfamily gene in maize, *narrow leaf 4* (*ZmNL4*), was cloned and demonstrated to control leaf width. Loss-of-function mutants exhibit significantly narrower leaves and reduced grain yield [31]. This finding identifies Kelch-ACBP proteins as important regulators of leaf morphology, yet whether their function is conserved in other cereals remains unknown.

In foxtail millet, the homologous gene of *ZmNL4* is *Seita.8G220000*, designated *SiNL4*. Sequence analysis revealed that SiNL4 shares 86.6% amino acid identity with ZmNL4 and similarly contains both a Kelch domain and an acyl-CoA-binding domain, suggesting a potentially conserved function. However, the role of *SiNL4* in leaf development and its contribution to yield-related traits have not been investigated. In this study, we performed CRISPR/Cas9-mediated knockout of *SiNL4* in foxtail millet to analyze its phenotypic effects on leaf width, vascular development, and grain yield. We also conducted cytological and transcriptomic analyses to explore the underlying molecular mechanisms. Furthermore, we demonstrated the functional conservation of *SiNL4* by expressing it in an *Arabidopsis thaliana* T-DNA insertion mutant of the homologous gene.

## 2. Materials and Methods

### 2.1. Plant Materials and Growth Condition

**Figure 1:**
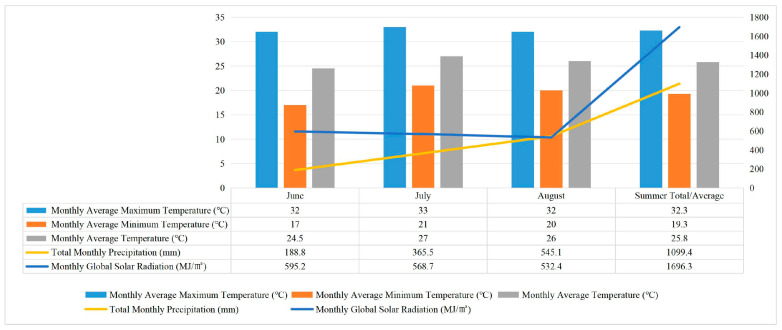
Summary of overall weather conditions for summer 2025 (June–August).

The *Sinl4* knockout lines were generated from the foxtail millet variety Ci846, which possesses high transformation efficiency. Two homozygous mutants with frame-shift mutations (*ko1* and *ko2*) were selected for further study. Ci846, *ko1*, and *ko2* were grown in the field under natural conditions during the summer of 2025 at the Shenfeng Experimental Station of Shanxi Agricultural University in Taigu District, Jinzhong City, Shanxi Province, China (37.400° N, 112.400° E). The weather conditions in the region during the summer of 2025 (June–August) are presented in Figure 1. For phenotypic evaluation of seedling root traits, seedlings were grown on MS medium in Petri plates for 10 days after germination.

Tobacco (*Nicotiana benthamiana*) seedlings used for subcellular localization assays were grown in the greenhouse under a 16 h light/8 h dark photoperiod at 22 °C. *Arabidopsis thaliana* plants, including the wild-type Col-0, the T-DNA insertion mutant *Atnl4* (SALK_004960, *AT5G18590*), and transgenic lines (overexpression lines OE1 and OE2; complementation lines COM1 and COM2), were grown in the greenhouse under the same conditions (16 h light/8 h dark, 22 °C, light intensity 15,000 Lx). Images of seeds and grains were captured using a stereomicroscope (M165FC, Leica, Wetzlar, Germany).

### 2.2. Vector Construction and Plant Transformation

To construct the CRISPR-Cas9 vector for the *SiNL4* gene, a single sgRNA targeting sequence within the 13th exon of *SiNL4* was designed using the web-based tool CRISPR-P 2.0 and inserted into the assembled into B*sa*I sites of the pHUE411 vector [32]. The generated construct was introduced into foxtail millet Ci846 by *Agrobacterium*-mediated transformation [33]. Transgenic plants were screened, and homozygous knockout lines were identified by PCR amplification and Sanger sequencing using primers flanking the off-target site.

To generate the overexpression vector p35S:*SiNL4*, the full-length CDS of *SiNL4* was amplified from first-strand cDNA of the foxtail millet cultivar Jingu21 using gene-specific primers containing K*pnI* and X*baI* restriction sites. The PCR product was purified using an agarose gel DNA recovery kit (DP209, Tiangen Biochemical Technology, Beijing, China) and inserted into the p35S:*GFP* vector (p35S-MCS-EGFP-6 × His-35S-Hyg) between the K*pnI* and X*baI* sites using the homologous recombination method. The recombinant plasmid was verified by sequencing using the primer p35S-SiNL4-P. The confirmed plasmid was extracted using a plasmid miniprep kit (DP103, Tiangen Biochemical Technology, Beijing, China) and transformed into *Agrobacterium* tumefaciens strain GV3101. The resulting *Agrobacterium* strains were used to transform wild-type *Arabidopsis thaliana* (Col-0) and the *Atnl4* mutant by the floral-dip method [33]. Transgenic plants were selected on hygromycin-containing medium. T1-positive plants were individually harvested; single-locus insertion lines showing a 3:1 segregation ratio on hygromycin selection were advanced to obtain T3 homozygous transgenic progeny for phenotypic analysis. All primers used for vector construction are listed in Table S1.

### 2.3. Phenotypic Evaluation

The leaf length and leaf width of Ci846, *ko1*, and *ko2* were measured at the heading stage in the field using a ruler. The plant height, peduncle length, panicle length, and panicle width were measured during the mature period. After harvest, the panicle weight, spikelet number, grain number per spikelet, grain length, grain width, grain perimeter, grain area, and 1000-grain weight were measured. Grain were captured using a stereomicroscope (BX51TF, Olympus, Tokyo, Japan) and quantified with ImageJ (v1.54g) software [34]. Seedling root length was measured in the greenhouse 10 days after germination. The *Arabidopsis* root length, plant height, rosette leaf length, rosette leaf width, silique length, number of seeds per silique, seed length, seed width, and 1000-seed weight were measured for Col-0, *Atnl4*, and the transgenic lines (OE1, OE2, COM1, COM2). Seedling root length was measured in the greenhouse 10 days after germination, rosette leaf traits were assessed 35 days after transplantation, and plant height and silique-related traits were scored at the mature stage.

### 2.4. RNA Extraction and Quantitative Reverse-Transcription PCR (qRT-PCR)

Total RNA was extracted from roots and shoots at the three-leaf stage, leaves and stems at the jointing stage, and leaves and young panicles at the booting stage of foxtail millet, as well as from rosette leaves of *Arabidopsis*, using the AGPC method [35]. Tissue samples (100–200 mg) were ground to a fine powder in liquid nitrogen, homogenized in cold AGPC extraction buffer, and extracted with chloroform. RNA was precipitated with an equal volume of isopropanol, washed twice with 70–75% ethanol, air-dried, and dissolved in RNase-free water. RNA concentration and integrity were assessed by spectrophotometry and 1% agarose gel electrophoresis. First-strand cDNA was synthesized using the StarScript Pro All-in-one RT Mix with gDNA Remover kit (Genstar, Beijing, China).

Quantitative real-time PCR was performed using 2× RealStar Universal SYBR qPCR Mix (Genstar, Beijing, China) on a real-time PCR system (CFX Duet, Bio-Rad, Hercules, CA, USA). *SiAct2* (*Seita.8G043100*) and *ACT2* (*AT3g18780*) were used as the internal reference gene in foxtail millet and *Arabidopsis*, respectively, and the assessment of each sample was conducted in three biological replicates. The relative expression levels were calculated using the 2^−ΔΔCt^ method [36]. All primers used for qRT-PCR are listed in Table S1.

### 2.5. Subcellular Localization

To investigate the subcellular localization of SiNL4, the full-length CDS of *SiNL4* was cloned into the p35S:*GFP* vector to generate the p35S:*SiNL4-GFP* fusion construct. The p35S:*SiNL4-GFP* and the control p35S:*GFP* constructs were separately introduced into *Agrobacterium* tumefaciens strain GV3101. For co-localization analysis, the p35S:*SiNL4-GFP* construct was co-infiltrated with mitochondrial and plasma membrane markers (Mito and PIP2, respectively) into the abaxial surface of leaves of six-week-old *Nicotiana benthamiana*. The *Agrobacterium* strains were resuspended in infiltration buffer (10 mM MgCl_2_, 10 mM MES, 100 μM acetosyringone, pH 5.5) to an OD_600_ of 0.4 before infiltration. After infiltration, plants were placed at 28 °C for 72 h. The GFP fluorescence signal was visualized using a laser scanning confocal microscope (FV3000, Olympus, Hachioji, Tokyo, Japan). Images were captured under identical settings for both the fusion protein and the GFP-only control. Mito indicates a mitochondrial marker, and PIP2 indicates a plasma membrane marker.

### 2.6. Phylogenetic Analysis and Protein Tertiary Structure Prediction

The protein sequence of ZmNL4 was used as a query to perform BLASTP (Phytozome v14) searches against the protein database of foxtail millet, rice, maize, and *Arabidopsis thaliana* in the Phytozome (https://phytozome-next.jgi.doe.gov/ (accessed on 7 February 2026)). High-confidence homologs (E-value ≤ 6.11 × 10^−31^) were retrieved (Table S2). The conserved domains of the retrieved proteins were analyzed using InterProScan (v5.77-108.0) and the NCBI Conserved Domain Database. Multiple sequence alignment was conducted using the ClustalW algorithm implemented in MEGA11 (v11.0.13) [37]. The phylogenetic tree was constructed using the neighbor-joining (NJ) method with the Poisson correction model, and branch support was evaluated with 1000 bootstrap replicates. Homology modeling of the three-dimensional structure of the target protein was performed using the SWISS-MODEL online platform (https://swissmodel.expasy.org (accessed on 7 February 2026)) [38].

### 2.7. Histological Analysis

All foxtail millet plants were grown in the field. At the heading stage, the widest portion of the flag leaf from Ci846, *ko1*, and *ko2* was sampled for histological analysis. For free-hand sectioning to measure epidermal cell dimensions, the leaf epidermal tissue was carefully peeled off using a sharp blade and placed on a glass slide. The sections were stained with 0.05% toluidine blue solution and photographed under a research-grade upright metallurgical microscope (BX51TF, Olympus, Hachioji, Tokyo, Japan). Digital images were captured at constant magnification, and cell length, cell width, and the number of cells per unit area in both transverse and longitudinal directions were measured using ImageJ (v1.54g) software.

For paraffin sectioning to examine vascular bundle morphology, flag leaf tissues were fixed in FAA fixative (70% ethanol, 5% acetic acid, and 3.7% formaldehyde, *v*/*v*) under vacuum at 4 °C for 16–18 h, followed by thorough rinsing with tap water for 24 h. To remove siliceous deposits, the fixed tissues were softened in 12% hydrofluoric acid for 72 h and then washed under running water for 48 h. Dehydration was performed through a graded ethanol series (70%, 80%, 95%, and three changes of 100% ethanol, 1.5 h each time), followed by clearing three times using xylene (1 h each time). The cleared samples were infiltrated with paraffin wax through a stepwise temperature gradient (35 °C to 70–75 °C) and finally embedded in molten paraffin. Transverse sections (12 μm thick) were cut using a rotary microtome (RM2126, Leica, Wetzlar, Germany), mounted on chrome alum gelatin-coated slides, and dried at 50 °C for 2 days. After deparaffinization in two changes of xylene and rehydration through a graded ethanol series, the sections were stained with 0.05% toluidine blue, dehydrated in absolute ethanol, cleared in xylene, and mounted with neutral gum. The stained sections were scanned using a Digital Pathology Slide Scanner (KF-SCAN-CL, Konfoong Bioinformation Tech. Co., Ltd., Yuyao, China), and leaf cross-section images were captured and analyzed using KFSlideOS 1.1.2.0 software. Large vascular bundles (LVBs) and small vascular bundles (SVBs) were identified and counted across the entire leaf width.

### 2.8. Measurement of Chlorophyll Content

Fresh flag leaves from Ci846, *ko1*, and *ko2* at the grain-filling stage were collected with three biological replicates. The chlorophyll content was measured according to previously established ethanol extraction methods [39,40]. Leaf samples (approximately 0.05 g) were cut into small pieces, precisely weighed, and placed in 15 mL centrifuge tubes containing 10 mL of 96% (*v*/*v*) ethanol. The tubes were stored in the dark at room temperature for three days. The absorbance of the resulting extract was measured at 665 nm, 649 nm, and 470 nm using a microplate spectrophotometer (Sunrise, Tecan, Grödig, Austria). The concentrations of chlorophyll a (Chl a), chlorophyll b (Chl b), total chlorophyll (Chl a + b), and total carotenoids (Car) were calculated using the equations established by Lichtenthaler and Wellburn [41]:

Chl a (μg/mL) = 13.95 A_665_ − 6.88 A_649_

Chl b (μg/mL) = 24.96 A_649_ − 7.32 A_665_

Total Chl = Chl a + Chl b

Car (μg/mL) = (1000 A_470_ − 2.05 × Chl a − 114.8 × Chl b)/245

The final pigment content was expressed as mg per gram of fresh weight.

### 2.9. Measurement of Photosynthetic Parameters

Photosynthetic parameters of the flag leaves of Ci846, *ko1*, and *ko2* were measured at three developmental stages: the heading stage, the grain-filling stage, and the physiological maturity stage. Measurements were conducted between 9:00 a.m. and 11:00 a.m. when the ambient light intensity exceeded 1000 μmol·m^−2^·s^−1^ using a portable photosynthesis analyzer CI-340 (CID Company, Roseville, CA, USA). The measured parameters encompassed net photosynthetic rate (*Pn*, μmol CO_2_ m^−2^ s^−1^), transpiration rate (*Tr*, mmol H_2_O m^−2^ s^−1^), stomatal conductance (*Gs*, mmol m^−2^ s^−1^), and intercellular carbon dioxide concentration (*Ci*, μmol mol^−1^). Each parameter was measured with 14 replicates, and measurements were performed on the fully expanded flag leaf of the main culm.

### 2.10. RNA-Seq

Shoot tissues of Ci846 and *ko1* at the seedling stage in the field were collected with three independent biological replicates for transcriptome analysis. Total RNA was extracted, and library construction and sequencing were performed by Shanghai OE Biotech Co., Ltd.(Shanghai, China). Raw sequencing reads were filtered to obtain high-quality clean reads. The clean reads were mapped to the foxtail millet reference genome. Gene expression levels were quantified as FPKM (Fragments Per Kilobase of transcript per Million mapped reads). The DESeq2 (v1.22.2) [42] method was employed to screen differentially expressed genes (DEGs) using the following criteria: |log_2_ Fold Change| > 1 and adjusted *p* value < 0.05. Functional annotation of the DEGs was performed by Gene Ontology (GO) enrichment analysis. To further understand the functional classification and expression patterns of DEGs, the identified DEGs were categorized according to functional annotation into groups related to leaf development were displayed using cluster heatmap analysis. The expression trends of several key candidate DEGs were further validated by qRT-PCR (Table 1).

## 3. Results

### 3.1. Identification and Phylogenetic Analysis of SiNL4

**Figure 2:**
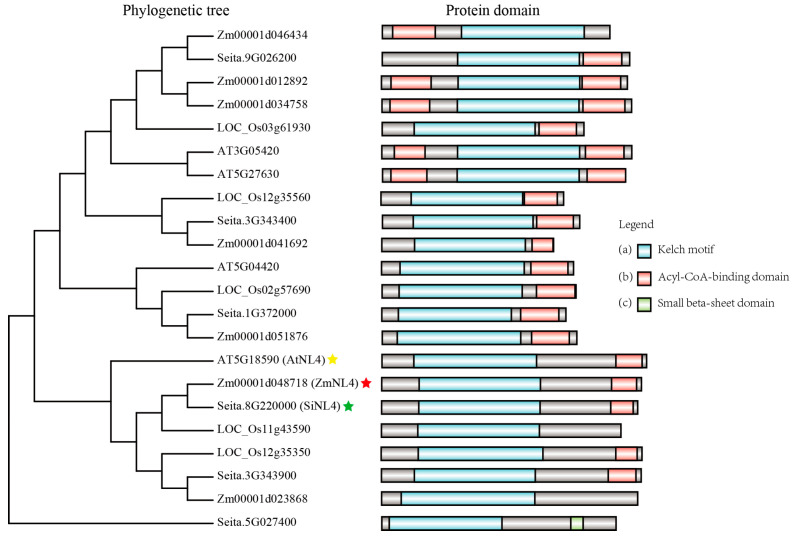
Phylogenetic tree for the SiNL4 protein. Sequences were aligned with ClustalX, and the N-J tree with 1000 bootstrap repetitions was calculated with MEGA11. The protein domains analyzed by InterProScan (v5.77-108.0) were shown at right of the tree. The green, red, and yellow pentacle represented SiNL4, ZmNL4, and AtNL4, respectively.

Our previous study shown that a Kelch motif containing protein ZmNL4 regulated leaf width and yield-related traits in maize [31]. To investigate the function of its homologous gene in foxtail millet, phylogenetic tree of ZmNL4 and its homologs from maize, rice, foxtail millet, and *Arabidopsis* was firstly constructed (Figure 2). We found that SiNL4 in foxtail millet and ZmNL4 belonged to the same clade (Figure 2) and shared an 86.6% amino acid identity (Figure S1, Table S2). Domain prediction revealed that SiNL4 contained two conserved protein domains: an N-terminal Kelch domain (residues 92–416) and a C-terminal acyl-CoA-binding domain (residues 613–673) (Figure 2). Tertiary structure prediction by homology modeling showed that these proteins all possessed the typical β-propeller structure characteristic of the Kelch family, and the spatial arrangement of SiNL4 was highly similar to that of ZmNL4 (Figure S2). The phylogenetic tree also showed that LOC_Os11g43590 in rice and AT5G18590 in *Arabidopsis thaliana* exhibited high amino acid similarity with ZmNL4; however, their functions in regulating leaf width have not yet been reported (Figure 2).

### 3.2. Knockout of the SiNL4 Gene Altered Leaf Width and Yield-Related Traits

To analyze the function of *SiNL4* in regulating leaf width, we conducted the genome editing of *SiNL4* in foxtail millet Ci846 using the CRISPR/Cas9 system. A single guide RNA targeting the 13th exon of *SiNL4* was transformed into Ci846 through *Agrobacterium*-mediated transformation, and two homozygous frame-shift mutants (*ko1* and *ko2*) were generated, which resulted in premature termination of protein translation (Figure S3A,B).

**Figure 3:**
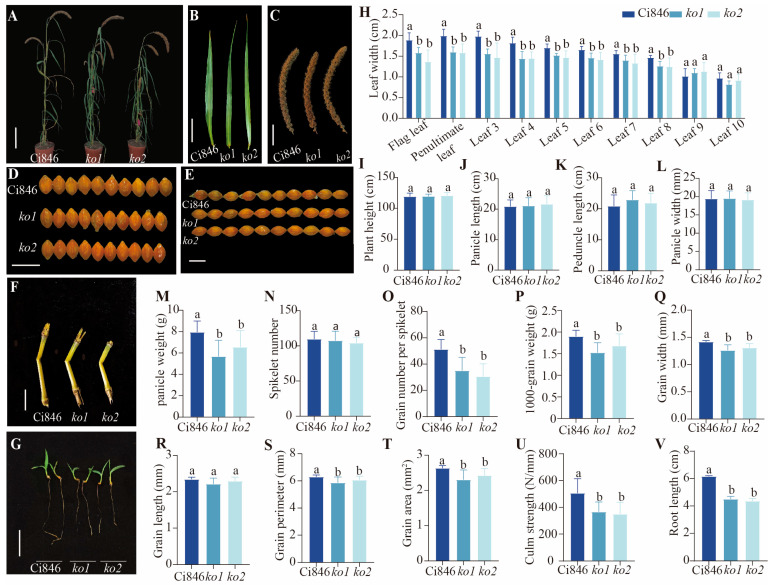
Phenotypic characterization of Ci846, *ko1* and *ko2.* (**A**) Ci846, *ko1*, and *ko2* plants grown in the field at the grain-filling stage. Scale bar = 20 cm. (**B**) Flag leaves of Ci846, *ko1*, and *ko2*. Scale bar = 5 cm. (**C**) Panicles of Ci846, *ko1*, and *ko2*. Scale bar = 5 cm. (**D**,**E**) Grain width (**D**) and grain length (**E**) of Ci846, *ko1*, and *ko2*. Scale bar = 3 mm. (**F**) Stems of Ci846, *ko1*, and *ko2*. Scale bar = 1 cm. (**G**) Root length of Ci846, *ko1*, and *ko2* seedlings. Scale bar = 2 cm. (**H**–**V**) Leaf width (**H**), plant height (**I**), panicle length (**J**), peduncle length (**K**), panicle width (**L**), panicle weight (**M**), spikelet number (**N**), grain number per spikelet (**O**), 1000-grain weight (**P**), grain width (**Q**), grain length (**R**), grain perimeter (**S**), grain area (**T**), stem rigidity (**U**), and root length (**V**) of Ci846, *ko1*, and *ko2*. Data were presented as means ± SD. Different letters above columns indicate significant differences (*p* < 0.05, one-way ANOVA, Tukey’s test, *n* = 15).

Phenotypic analysis at the heading and mature stages revealed significant differences between the wild-type Ci846 and the knockout lines. Compared with Ci846, *ko1* and *ko2* showed significantly narrower leaves from the flag leaf to the eighth leaf below the flag leaf (Figure 3B,H). However, no significant differences were observed in plant height, panicle length, peduncle length, or panicle width (Figure 3A,C,I–L). At the mature stage, the panicle weight, grain number per spikelet, and seed setting rate of *ko1* and *ko2* were significantly reduced compared with Ci846 (Figure 3M–O). In terms of grain size, grain width, grain perimeter, and grain area were all significantly decreased in *ko1* and *ko2*, whereas grain length showed no significant difference (Figure 3Q–T). Consequently, the 1000-grain weight of *ko1* and *ko2* was dramatically reduced (Figure 3P). These results indicated that the reduction in panicle weight in the mutants was mainly attributable to decreased seed setting rate and lower 1000-grain weight. Taken together, these results demonstrated that the knockout of *SiNL4* negatively affected leaf width and yield-related traits. Moreover, the stem rigidity of *ko1* and *ko2* was markedly lower than that of Ci846 (Figure 3F,U), and the seedling root length was significantly shorter than that of Ci846 10 days after germination (Figure 3G,V).

### 3.3. SiNL4 Influenced Leaf Width by Regulating Cell Expansion and the Number of SVB

**Figure 4:**
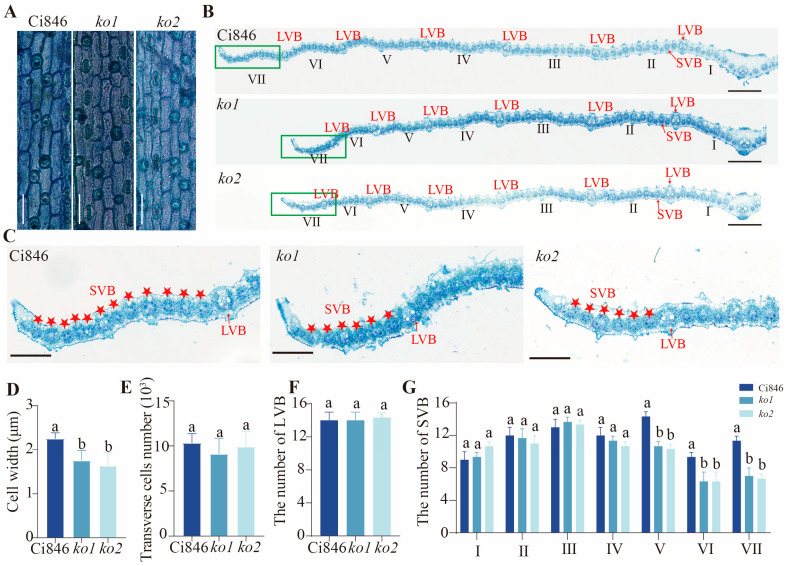
Analysis of leaf tissue cell structure. (**A**) Part of a longitudinal section of Lower epidermis of flag leave of Ci846, *ko1*, and *ko2*. Scale bar = 10 μm. (**B**) Part of a cross section of flag leave of Ci846, *ko1*, and *ko2*. LVB: large vascular bundle. SVB: small vascular bundle. Roman numerals indicate the regions between two adjacent LVBs. Scale bar = 500 μm. (**C**) Magnified view of the region indicated by the green box in (**B**). Red arrow indicates LVB, and red asterisks indicate SVB. Scale bar = 200 μm. (**D**) Epidermal cell width and (**E**) number of transverse cells. (**F**) Number of LVBs and (**G**) number of SVBs. Data were presented as means ± SD. Different letters above columns indicate significant differences (*p* < 0.05, one-way ANOVA, Tukey’s test, *n* = 5).

To clarify the cellular basis of the narrower leaf phenotype in the knockout lines, histological analysis of flag leaves from Ci846, *ko1*, and *ko2* was first performed using free-hand sectioning at the heading stage (Figure 4A). Compared with Ci846, the epidermal cell width of *ko1* and *ko2* were significantly reduced (Figure 4D). When counting cell numbers, we found no significant difference in the number of transverse cells (Figure 4E). This indicated that the cell expansion in the leaf epidermal tissue of *ko1* and *ko2* was largely compromised.

To more precisely examine the cause of the narrow leaf phenotype, we further observed the transverse sections of leaf blades by paraffin sectioning. It was found that the average number of large vascular bundles (LVBs) in *ko1* and *ko2* was comparable to that in Ci846 (Figure 4B,C,F). However, the average number of small vascular bundles (SVBs) among the large vascular bundles near the leaf edge was reduced in *ko1* and *ko2* (Figure 4B,C,G). Overall, these results indicated that *SiNL4* might affect leaf width by regulating cell expansion along the leaf-width axis and the number of SVB close to leaf edge.

### 3.4. The Spatiotemporal Expression Analysis and Subcellular Localization of SiNL4

**Figure 5:**
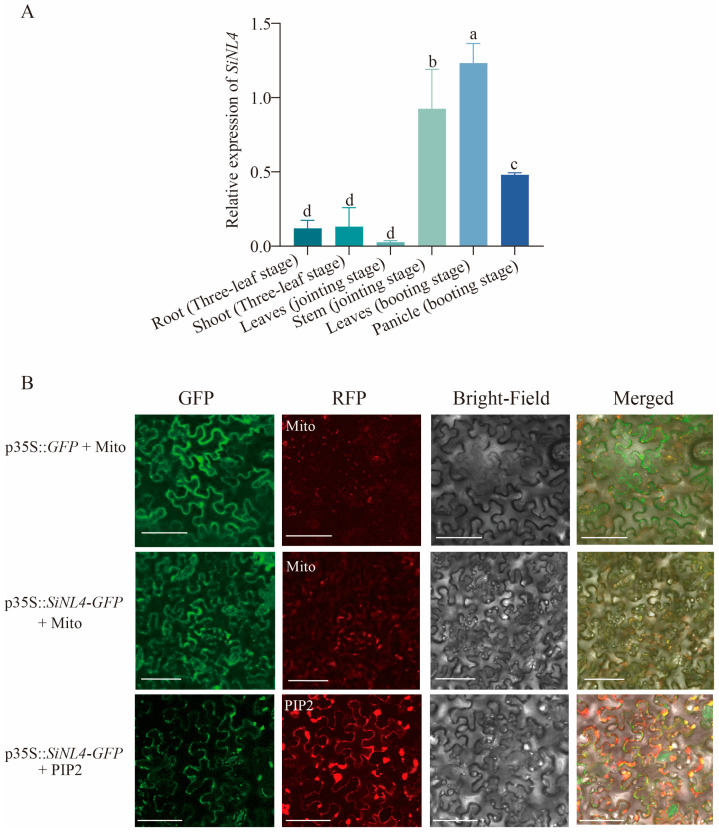
The spatiotemporal expression analysis and subcellular localization of *SiNL4*. (**A**) The relative expression levels of *SiNL4* in different tissues of the foxtail millet variety Jingu 21 at different developmental stages. Data were presented as means ± SD. Different letters above columns indicate significant differences (*p* < 0.05, one-way ANOVA, Tukey’s test, *n* = 3). (**B**) Subcellular localization of SiNL4 protein in 6-week-old *Nicotiana benthamiana* leaves; *GFP* was used as a control. Mito indicates a mitochondrial marker, and PIP2 indicates a plasma membrane marker.

To analyze the expression pattern of SiNL4, reverse transcription-quantitative PCR (RT-qPCR) was performed using RNA extracted from different tissues of the foxtail millet cultivar Jingu 21 at various developmental stages. The results showed that *SiNL4* was expressed in multiple tissues. At the three-leaf stage, expression levels in the roots and shoots were relatively low. At the jointing stage, expression in the leaves remained low, whereas expression in the stems was relatively high. At the booting stage, high expression levels were detected in both the leaves and the young panicle (Figure 5A). These results indicated that *SiNL4* was preferentially expressed in stems, leaves, and young panicles, tissues that are actively undergoing cell expansion and morphogenesis.

To determine the subcellular localization of SiNL4, the coding sequence of SiNL4 was fused to GFP and transiently co-expressed with mitochondrial (Mito) and plasma membrane (PIP2) markers in *Nicotiana benthamiana* leaves. The control GFP signal was distributed throughout the entire epidermal cell, including the nucleus and cytoplasm, whereas the p35S::*SiNL4*-*GFP* fusion protein signal was predominantly detected in the mitochondria and plasma membrane (Figure 5B).

### 3.5. Ectopic Expression of SiNL4 Restored the Phenotypes of Atnl4

**Figure 6:**
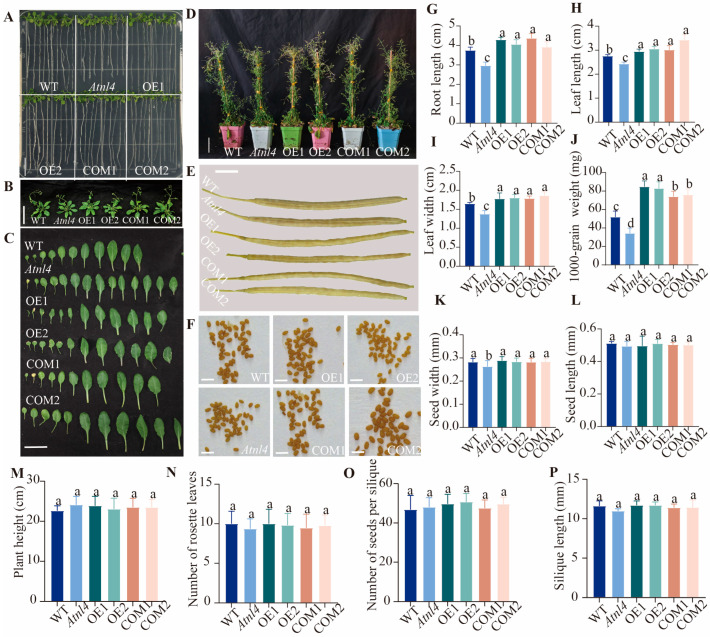
Phenotypic characterization of *Arabidopsis thaliana* Col-0, *Atnl4*, overexpression lines, and complementation lines. (**A**) Seedling root length. Scale bar, 1 cm. (**B**) Plants at 28 days after transplantation. Scale bar, 6 cm. (**C**) Rosette leaves at 35 days after transplantation. Scale bar, 6 cm. (**D**) Mature plants. Scale bar, 5 cm. (**E**) Mature siliques. Scale bar, 2 mm. (**F**) Mature seeds. Scale bar, 1 mm. (**G**–**P**) Quantitative analysis of root length (**G**), rosette leaf length (**H**), rosette leaf width (**I**), 1000-seed weight (**J**), seed width (**K**), seed length (**L**), plant height (**M**), rosette leaf number (**N**), number of seeds per silique (**O**), and silique length (**P**). Data were presented as means ± SD. Statistical analyses were performed using Tukey’s multiple comparisons test. Different letters indicate significant differences at *p* < 0.05.

To characterize the function of *SiNL4* in *Arabidopsis*, we first obtained a T-DNA insertion line of *AtNL4* (*AT5G18590*, homolog of *SiNL4*, Figure 6), firstly. Phenotypic identification showed that compared with wild-type Col-0, *Atnl4* plants exhibited significantly shorter root length, smaller rosette leaves, reduced seed width, and decreased 1000-seed weight. However, there were no significant differences in plant height, rosette leaf number, silique length, number of seeds per silique, or seed length (Figure 6).

To validate the function of *SiNL4* in regulating leaf width, we generated stable overexpression lines of *SiNL4* in the Col-0 background (OE1 and OE2) and complementation lines in the *Atnl4* mutant background (COM1 and COM2) using the CaMV35S promoter. RT-qPCR analysis confirmed that *SiNL4* was highly expressed in both the overexpression and complementation lines, with the expression levels in OE1 and OE2 being higher than those in COM1 and COM2 (Figure S4). Phenotypic analysis demonstrated that *SiNL4* not only restored the grain width of *Atnl4*, but also resulted in significant increases in root length, rosette leaf length and width, and 1000-seed weight in the OE1, OE2, COM1, and COM2 lines compared to both *Atnl4* and Col-0 (Figure 6G–K). The plant height, silique length, number of seeds per silique, and seed length were comparable among Col-0, *Atnl4*, and the transgenic lines (Figure 6L–P). Taken together, these results demonstrated that *SiNL4* complemented the phenotype of *Atnl4* and that the function of *SiNL4* and *AtNL4* in regulating leaf width is highly conserved between monocots and dicots.

### 3.6. SiNL4 Regulated the Photosynthetic Capacity

**Figure 7:**
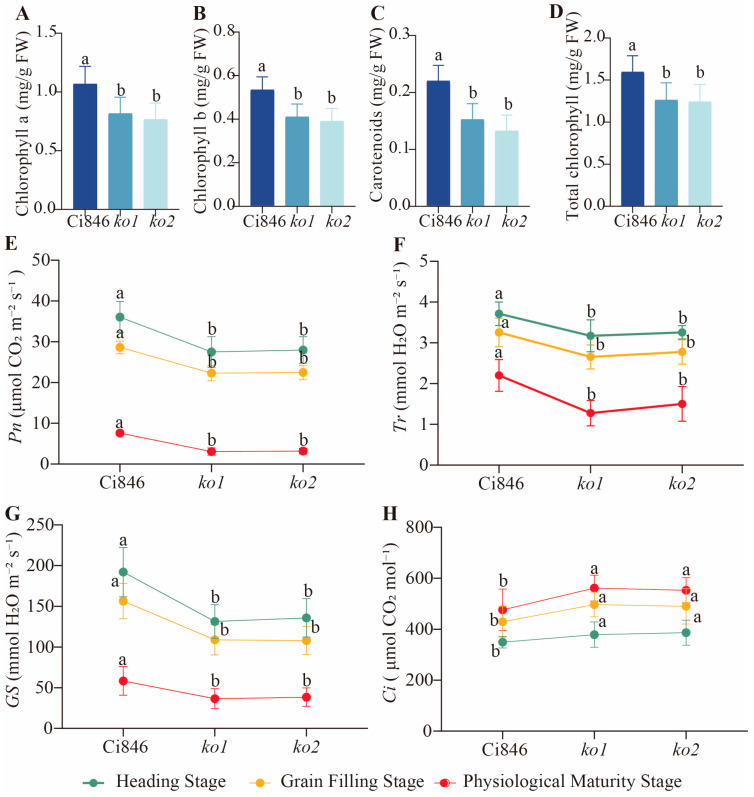
Analysis of chlorophyll content and photosynthetic capacity in Ci846, *ko1*, and *ko2*. (**A**) Chlorophyll a content in Ci846, *ko1* and *ko2*. (**B**) Chlorophyll b content in Ci846, *ko1* and *ko2*. (**C**) Carotene content in Ci846, *ko1* and *ko2*. (**D**) Total chlorophyll content in Ci846, *ko1* and *ko2*. (**E**) Net photosynthetic rate of Ci846, *ko1* and *ko2*. (**F**) Transpiration rate of Ci846, *ko1* and *ko2*. (**G**) Stomatal conductance of Ci846, *ko1* and *ko2*. (**H**) Intercellular CO_2_ concentration of Ci846, Ci846, *ko1* and *ko2*. Data were means ± SD. Statistical analyses were performed using Tukey’s multiple comparisons test. Different letters indicated significant differences at *p* < 0.05.

In addition to the narrow leaf phenotype, we observed that the leaf color of *ko1* and *ko2* was slightly yellower than that of Ci846 at the heading stage. Given that chlorophyll content is closely related to photosynthetic capacity, we measured the chlorophyll and carotenoid contents in the flag leaves of Ci846, *ko1*, and *ko2* at the grain-filling stage. The results showed that the contents of chlorophyll a, chlorophyll b, carotenoids, and total chlorophyll in *ko1* and *ko2* were all significantly lower than those in Ci846 (Figure 7A–D).

Photosynthetic parameters were further measured at the heading stage, grain-filling stage, and physiological maturity stage. Across all three developmental stages, the net photosynthetic rate (*Pn*), transpiration rate (*Tr*), and stomatal conductance (*Gs*) of *ko1* and *ko2* were significantly reduced compared with Ci846, whereas the intercellular CO_2_ concentration (*Ci*) was significantly increased (Figure 7E–H). The decrease in *Pn* accompanied by an increase in *Ci* indicated that the reduction in photosynthetic capacity was not primarily caused by stomatal limitation, but rather by impaired photosynthetic activity of mesophyll cells. These results suggested that *SiNL4* might be involved in regulating photosynthesis.

### 3.7. Differential Gene Expression of Ci846 and ko1

**Figure 8:**
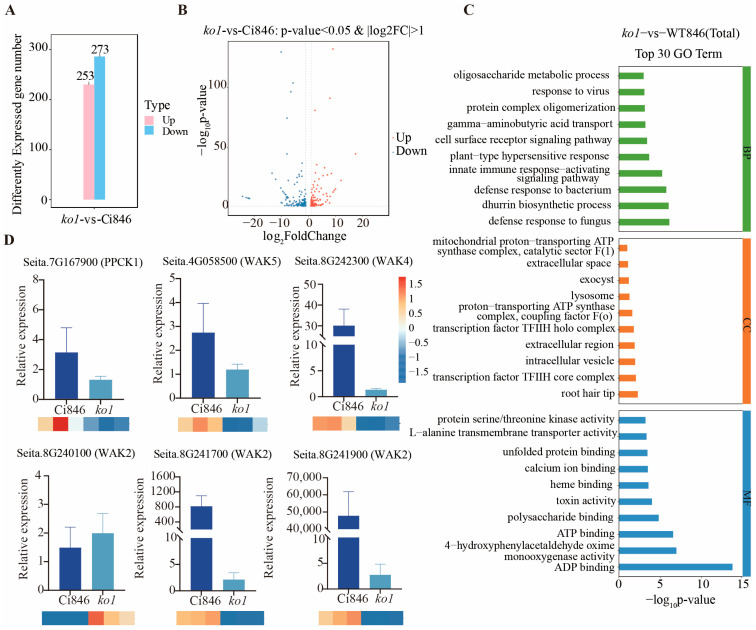
RNA-seq analysis of foxtail millet *ko1* and Ci846. (**A**) The number of upregulated and downregulated genes in *ko1* compared with Ci846. (**B**) The volcano plot showed the distribution of differentially expressed genes (DEGs). Red dots and blue dots represented upregulated and downregulated genes in *ko1* compared with Ci846, respectively. Gray dots represented genes where the expression level between *ko1* and Ci846 was not significant. (**C**) GO enrichment analysis. (**D**) RT-qPCR analysis of genes related to the development of leaves. The error bar represents the standard deviation of the three bioreplicates. Significant differences between samples of statistical data are indicated by different letters placed above the bar chart (determined by Student’s *t*-test with *p*  <  0.05).

To gain deeper insights into the function of *SiNL4* in regulating leaf width, transcriptome sequencing was performed using shoot tissues of Ci846 and *ko1* at the seedling stage. Data analysis revealed a total of 526 differentially expressed genes (DEGs) between Ci846 and *ko1* (|log_2_ Fold Change| > 1, *p* value < 0.05) (Table S3). Compared with Ci846, 273 genes were significantly downregulated and 253 genes were significantly upregulated in *ko1* (Figure 8A,B). Gene Ontology (GO) enrichment analysis revealed that the upregulated genes were significantly enriched in biological processes related to defense responses and secondary metabolism, including “dhurrin biosynthetic process”, “innate immune response-activating signaling pathway”, “defense response to fungus”, “defense response to bacterium”, and “plant-type hypersensitive response” (Figure 8C, Table S4). In contrast, the downregulated genes were mainly enriched in processes related to carbohydrate metabolism and cell development, including “cell surface receptor signaling pathway”, “root hair cell tip growth” “sucrose catabolic process”, “starch catabolic process”, and “oligosaccharide metabolic process” (Figure 8C, Table S5).

Among the DEGs, we found that the expression level of several genes positively regulating leaf development showed significantly decreased in *ko1* (Figure 8D). For example, *Seita.3G164800* encoded a type XI myosin, and the expression level of this gene was significantly reduced in *ko1*. Previous study has shown that loss of function of type XI myosins led to a significant reduction in the leaf width of the rosette leaves and a significant decrease in the size of the leaf epidermal cells in *Arabidopsis* [43]. Additionally, *Seita.7G167900*, a homolog of *Arabidopsis* phosphoenolpyruvate carboxylase kinase 1 (*PPCK1*), was also significantly downregulated in the *ko1*. It was reported that *PPCK1* in *Arabidopsis* was mainly expressed in the rosette leaves, and the loss-of-function mutant *ppck1* exhibited growth retardation and a significant reduction in the diameter of the rosette leaves [44]. Furthermore, the protein products of several genes including *Seita.4G058500*, *Seita.8G179700*, *Seita.8G240000*, *Seita.8G240100*, *Seita.8G241700*, *Seita.8G241900*, and *Seita.8G242300* were annotated as Wall-associated receptor kinase (WAK) and the expression level of these genes were downregulated in *ko1* except *Seita.8G240100*. *TaWAK2* was reported to regulate wheat leaf width through TaWAK2-TaNAL1-TaDST pathway and reduced protein stability of TaWAK2-A^A525V^ leading to a narrow leaf phenotype in wheat [7]. To validate the reliability of the RNA-seq data, six representative DEGs were selected for qRT-PCR verification (Figure 8D), including *WAK5* (*Seita.4G058500*), *PPCK1* (*Seita.7G167900*), *WAK2* (*Seita.8G241700*, *Seita.8G241900*), *WAK4* (*Seita.8G242300*), and *WAK2* (*Seita.8G240100*). The expression trends of all six genes detected by qRT-PCR were highly consistent with the RNA-seq results, confirming the accuracy of the transcriptomic data.

## 4. Discussion

### 4.1. SiNL4 Conservatively Regulates Leaf Width via Cell Expansion and Small Vascular Bundle Development

In our previous research, knockout of the maize gene *ZmNL4*, which encodes a Kelch-repeat domain protein, significantly reduced leaf width [31]. Here, we discovered that the homologous gene *SiNL4* in foxtail millet and *AtNL4* in *Arabidopsis thaliana* also exhibited narrow-leaf phenotypes in their respective mutants (Figure 2). Moreover, ectopic expression of *SiNL4* not only restored the rosette leaf width and grain width phenotypes of *Atnl4*, but also significantly increased the 1000-seed weight compared with wild-type Col-0 (Figure 6). This result strongly demonstrates that the function of *SiNL4* in regulating leaf width is conserved between monocotyledonous and dicotyledonous plants. However, an interesting difference was observed at the cellular level: *SiNL4* mainly affected leaf width by regulating epidermal cell expansion (Figure 4), whereas *ZmNL4* was reported to influence leaf width by affecting cell division [31]. Despite their conserved function in leaf width regulation, the underlying cellular mechanisms of *SiNL4* and *ZmNL4* may have diverged during evolution. This also highlights the functional diversity of Kelch family members in regulating leaf morphogenesis.

Our cytological analysis provides a clear cellular basis for the narrow-leaf phenotype of *SiNL4* knockouts. The free-hand sectioning data demonstrated that epidermal cell width in *ko1* and *ko2* was significantly reduced, whereas the number of transverse cells remained unchanged (Figure 4). This indicates that *SiNL4* specifically affects lateral cell expansion rather than cell proliferation in the leaf-width direction. The paraffin section results further revealed that the number of small vascular bundles (SVBs) near the leaf margin was markedly decreased in the mutants, whereas the number of large vascular bundles (LVBs) was unaffected (Figure 4). These findings suggest a dual regulatory pathway: *SiNL4* promotes leaf width by enhancing epidermal cell expansion on one hand, and by supporting the formation of SVBs in the marginal leaf regions on the other. Such a dual pathway has been documented in rice, where the narrow-leaf mutant *nal1* exhibits reduced leaf width accompanied by both decreased small vein number and diminished epidermal cell size [10,45]. The reduction in SVBs may compromise water and nutrient transport to the leaf margins [46], thereby indirectly limiting cell expansion capacity. The decrease in cell width and the reduction in SVB number appear to be complementary contributors to the final narrow-leaf phenotype, consistent with the developmental coupling between leaf vasculature and leaf shape observed in other species [47,48].

### 4.2. SiNL4 Knockout Impairs Photosynthetic Capacity and Yield, with Potential Agronomic Trade-Offs

Beyond the narrow-leaf phenotype, *ko1* and *ko2* plants displayed pale-yellow leaf color at the heading stage, and quantitative measurements confirmed that chlorophyll a, chlorophyll b, carotenoid, and total chlorophyll contents were significantly reduced (Figure 7). Photosynthetic gas exchange measurements across three developmental stages revealed that *Pn*, *Tr*, and *Gs* were significantly decreased in the mutants, while *Ci* was significantly elevated (Figure 7). A decrease in *Pn* coupled with an increase in *Ci* is a classic indicator of non-stomatal limitation, suggesting that the photosynthetic defect arises primarily from impaired mesophyll cell function rather than from restricted CO_2_ entry through stomata [49]. This conclusion is strongly supported by the transcriptome data, which showed that several genes involved in chloroplast development and function, including *CV*, *PTAC6*/*PAP8*, *DJC76*, and ATP synthase subunit genes, were significantly downregulated in *ko1*. The upregulation of Rubisco activase genes (*RCA1* and *RCAB*) in *ko1* may represent a compensatory response aimed at activating the remaining Rubisco to maintain basic carbon fixation capacity under conditions of chloroplast impairment, as RCA is known to be a multiple stress-responsive factor whose upregulation can help sustain CO_2_ assimilation under stress conditions [50,51]. It should be noted that the observed reductions in chlorophyll content and photosynthetic parameters may reflect both direct and indirect consequences of *SiNL4* loss of function. On one hand, the narrower leaf phenotype and altered vascular patterning could indirectly affect gas exchange by modifying mesophyll cell arrangement or internal leaf architecture, which in turn influences CO_2_ diffusion and light capture [52]. On the other hand, the downregulation of chloroplast development-related genes provides molecular evidence that *SiNL4* may directly influence photosynthetic function. Therefore, further experiments such as tissue-specific expression analysis or chloroplast isolation are needed to determine whether *SiNL4* plays a direct role in regulating photosynthesis.

Consistent with the impaired photosynthetic capacity, *SiNL4* knockout resulted in significant reductions in panicle weight, grain width, and thousand-grain weight (Figure 6), establishing *SiNL4* as a positive regulator of yield-related traits. However, it is worth considering whether the narrow-leaf architecture conferred by *SiNL4* knockout or knockdown could offer potential benefits under specific agronomic conditions. In several crops, moderately narrow leaves have been associated with improved canopy light penetration and increased planting density tolerance, which can lead to higher population-level yield despite reduced individual plant performance [53]. Additionally, narrower leaves with reduced transpiration area may enhance drought tolerance by decreasing water loss under water-limited conditions [54], which is particularly relevant for foxtail millet as a crop predominantly cultivated in arid and semi-arid regions. Therefore, while *SiNL4* functions as a positive regulator of leaf width and grain size, the *SiNL4* locus may offer a potential target for fine-tuning leaf architecture to balance individual plant productivity and population-level adaptability. Future studies evaluating the performance of *SiNL4* mutant or edited lines under varying planting densities and drought stress conditions will be necessary to fully assess its breeding application value.

### 4.3. SiNL4 May Coordinate Lipid Metabolism and Protein Degradation at Membrane Sites to Regulate Leaf Development

The protein encoded by *SiNL4* contains an N-terminal Kelch-repeat domain and a C-terminal acyl-CoA-binding (ACBP) domain. The Kelch-repeat domain forms a β-propeller structure that functions as a protein–protein interaction platform and, in multiple F-box proteins, acts as a substrate-recognition subunit to mediate ubiquitination and subsequent degradation of target proteins [28,55,56]. The ACBP domain is involved in acyl-CoA binding and transport, and in *Arabidopsis*, the close homologs AtACBP4 and AtACBP5 participate in lipid metabolism by binding oleoyl-CoA, with *acbp4* knockout leading to significant decreases in multiple membrane lipids [57,58]. Notably, SiNL4 lacks any known DNA-binding domain, and our subcellular localization experiment confirmed that SiNL4 is predominantly localized in the mitochondria and plasma membrane (Figure 5B), ruling out its direct action as a transcription factor. Instead, this dual membrane localization, together with its dual-domain architecture, suggests that SiNL4 may function as an integrator coupling lipid metabolism with protein degradation at membrane sites.

The ACBP domain facilitates the delivery of long-chain acyl-CoA esters to the plasma membrane, where they serve as essential substrates for membrane phospholipid synthesis and as precursors for cuticular lipid formation [59,60]. Plasma membrane lipid homeostasis is critical for membrane fluidity, vesicle trafficking, and the delivery of cell wall components—processes intimately linked to turgor-driven cell expansion [61]. Disruption of this lipid supply in *SiNL4* knockout plants could therefore impair membrane remodeling required for cell expansion, providing a mechanistic explanation for the reduced epidermal cell width observed in *ko1* and *ko2*. Concurrently, the Kelch-repeat domain recruits specific target proteins at the plasma membrane for ubiquitin-mediated degradation, including negative regulators of cell expansion and components of defense signaling.

This functional coupling model is supported by our transcriptomic data. Several genes known to positively regulate leaf width were significantly downregulated in *ko1*, including the type XI myosin gene, the *PPCK1* homolog, and multiple *WAK* genes (Figure 8D). The coordinated downregulation of these genes suggests that *SiNL4* may function upstream of them, potentially by targeting their transcriptional repressors for ubiquitin-dependent degradation. Additionally, the “phenylpropanoid biosynthesis” pathway showed bidirectional regulation, and “polysaccharide binding” and “calcium ion binding” functions were significantly enriched among downregulated genes, consistent with impaired cell wall modification. Although the identity of direct *SiNL4*-interacting proteins and the specific acyl-CoA substrates of its ACBP domain remain to be experimentally determined, this integrated model provides a testable framework for future studies combining co-immunoprecipitation, lipid-binding assays, and lipidomic profiling.

### 4.4. SiNL4 Mutation May Have Triggered a Growth-Defense Trade-Off at the Transcriptional Level

To gain deeper insight into the molecular function of *SiNL4*, we performed RNA-seq analysis. The transcriptomic profile of *ko1* exhibited a characteristic “growth-defense trade-off”: genes involved in defense responses (e.g., “defense response to fungus”, “innate immune response-activating signaling pathway”, “plant-type hypersensitive response”) and secondary metabolism (e.g., “dhurrin biosynthetic process”, “phenylpropanoid biosynthesis”) were significantly upregulated, whereas genes involved in carbohydrate metabolism (e.g., “sucrose catabolic process”, “starch catabolic process”) and cell development (e.g., “root hair cell tip growth”) were significantly downregulated. This transcriptional reprogramming suggests that the loss of *SiNL4* function causes plants to allocate limited resources away from basic growth and carbon assimilation toward defense and stress responses. Such a trade-off has been well documented in plants: when defense pathways are constitutively activated, growth is often suppressed due to resource reallocation and antagonistic crosstalk between hormone signaling pathways. The coordinated upregulation of ethylene-responsive factors (*RAP2-3*, *ERF012*), the auxin-responsive gene *IAA9*, WRKY transcription factors (*WRKY50*, *WRKY45-1*), and other stress-related genes further supports the notion that multiple hormone signaling networks are activated in *ko1*, likely contributing to both the initiation of defense responses and the suppression of leaf growth.

## 5. Conclusions

In summary, this study systematically characterized the function of the Kelch-repeat superfamily gene *SiNL4* in foxtail millet for the first time. We demonstrated that *SiNL4* positively regulates leaf width by modulating lateral cell expansion of epidermal cells and the development of small vascular bundles near the leaf margin, and its knockout significantly reduced yield-related traits including panicle weight, grain width, and 1000-grain weight. *SiNL4* knockout also impaired photosynthetic capacity, as evidenced by decreased chlorophyll content and reduced net photosynthetic rate accompanied by elevated intercellular CO_2_ concentration, indicating non-stomatal limitation. Transcriptomic analysis revealed that loss of *SiNL4* function led to extensive transcriptional reprogramming, characterized by upregulation of genes enriched in defense response and secondary metabolism pathways and downregulation of genes involved in carbohydrate metabolism and cell growth processes, including several known positive regulators of leaf width such as a type XI myosin gene, *PPCK1*, and multiple *WAK* genes. Ectopic expression of *SiNL4* in *Arabidopsis thaliana* rescued the *Atnl4* mutant phenotypes and significantly increased 1000-seed weight compared to Col-0, demonstrating that the regulatory function of this gene is conserved across monocots and dicots. Although SiNL4 likely functions through the ubiquitin–proteasome pathway rather than as a direct transcription factor, its direct substrates and the biochemical role of its ACBP domain remain to be elucidated. Collectively, this study provides a valuable candidate gene and theoretical basis for the genetic improvement of plant architecture and grain yield in foxtail millet and potentially other cereal crops.

## Figures and Tables

**Table 1:** List of primer sequences for several key candidate DEGs.

Gene Name	Forward Primer (5′-3′)	Reverse Primer (5′-3′)
*Seita.4G058500*	TGGCACTAGCTGTGGGTTCA	GTATTTCCAACAGCATGGCGG
*Seita.7G167900*	CTGATGCGCCGGATGATGTG	AGGTGGTGCAGCCGACCTGA
*Seita.8G240100*	CAGTACATGGAGTGCCTTGACAT	GAGCTCATGCTATCACCTGAGT
*Seita.8G241700*	GCTGCTGCAGTACTAGCAAC	CTGACGAAGAGCAGAGAGTTG
*Seita.8G241900*	ACTCGCATGGTGATCACGACT	CAGCAGATATGAGCTACGTGG
*Seita.8G242300*	CGTGTCCATTGCTAGCAGGT	TGCAACAGCCTCCCCATGAA

## Data Availability

The original contributions presented in this study are included in the article/Supplementary Material. Further inquiries can be directed to the corresponding authors.

## Supplementary Materials

The following supporting information can be downloaded at: https://www.mdpi.com/article/10.3390/plants15121826/s1. Figure S1: Protein sequences alignment of SiNL4 and ZmNL4; Figure S2: Prediction of the tertiary structure of SiNL4 and its homologous proteins; Figure S3: Sequence alignment of Ci846, *ko1*, and *ko2* and comparison of their protein structures; Figure S4: Relative expression levels of *SiNL4* in *Arabidopsis* Col-0, *Atnl4*, overexpression lines, and complementation lines; Table S1: Primers used in this study; Table S2: The protein sequences used for phylogenetic analysis; Table S3: List of differentially expressed genes between the Ci846 and *ko1*; Table S4: Gene ontology (GO) analysis of up-regulating genes in *ko1* compared with Ci846; Table S5: Gene ontology (GO) analysis of down-regulating genes in *ko1* compared with Ci846.
