# Supplementary material for: The Kelch-Repeat Superfamily Gene SiNL4 Regulates the Leaf Width in Foxtail Millet
Source: Plants (Basel). 2026 Jun 12;15(12):1826. doi: 10.3390/plants15121826 (PMC13306321; doi:10.3390/plants15121826)
Supplement: Supplementary file 1 [file plants-15-01826-s001.zip › Supplementary Data.pdf]

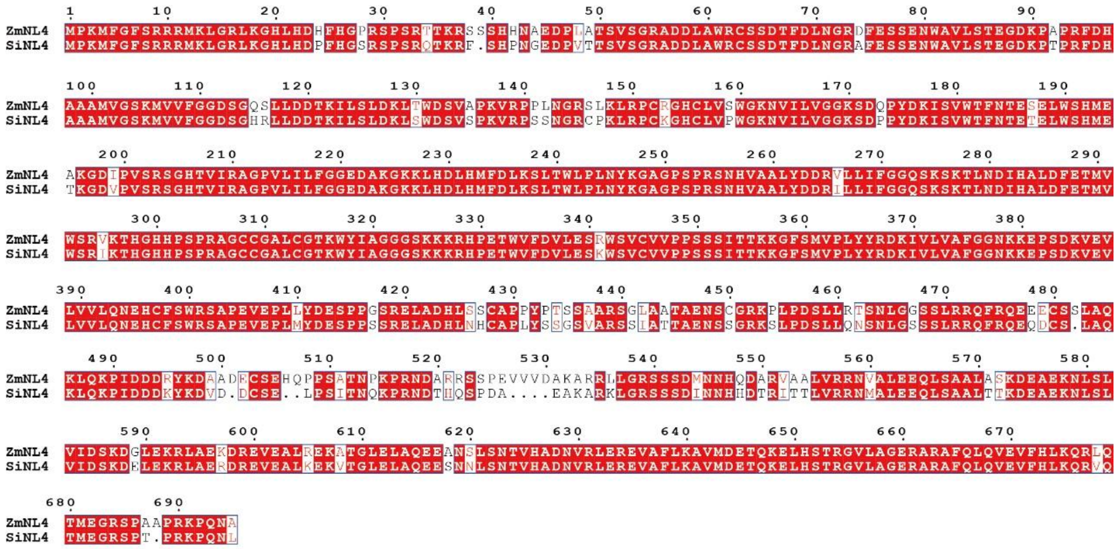

Figure S1 Protein sequences alignment of SiNL4 and ZmNL4. The alignment was generated with ClustalX using default parameters and modified using the ESPript 3.2 server.

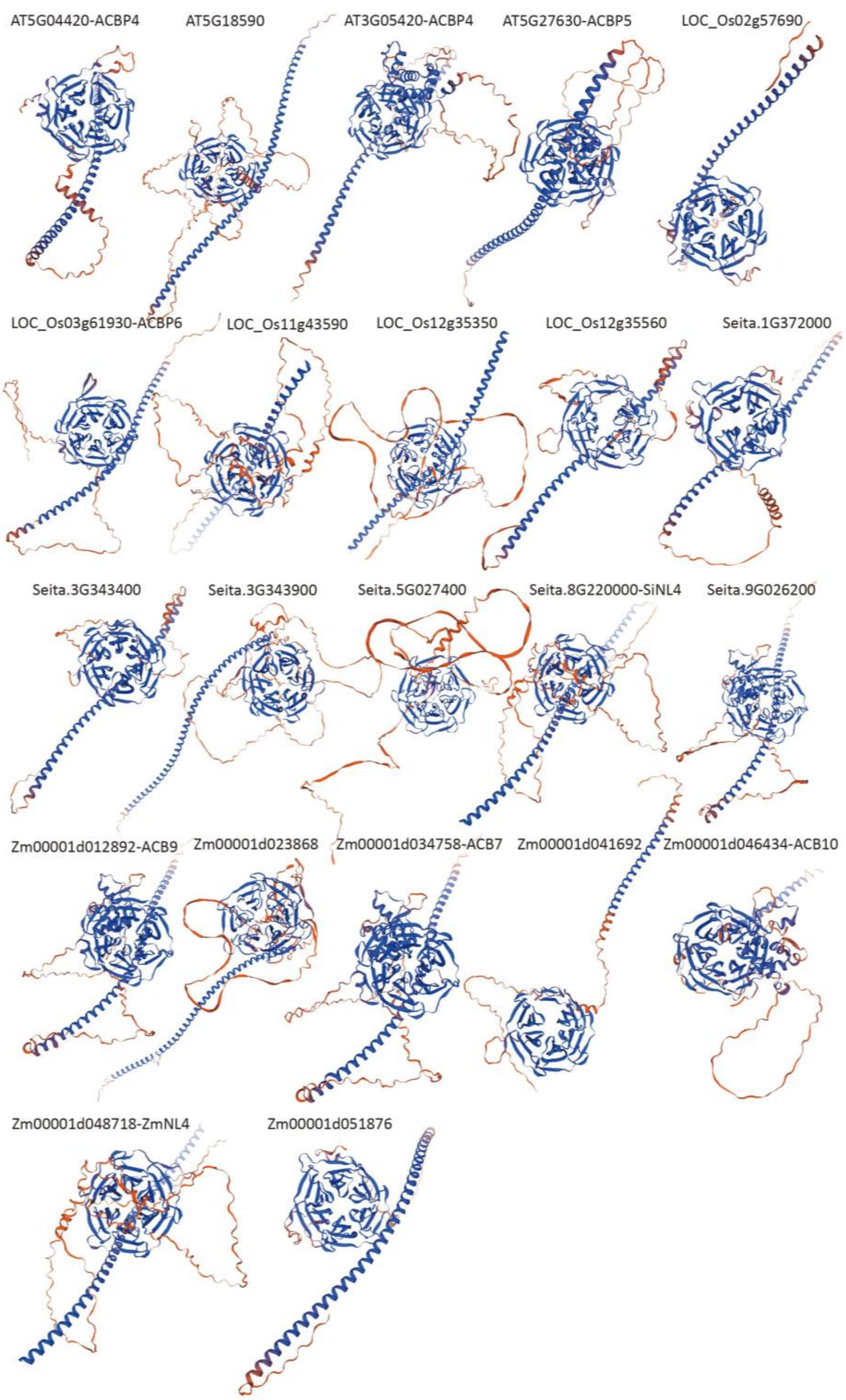

Figure S2 Prediction of the tertiary structure of SiNL4 and its homologous proteins. The tertiary structures of SiNL4 and its homologous proteins were predicted using the SWISS-MODEL online tool.

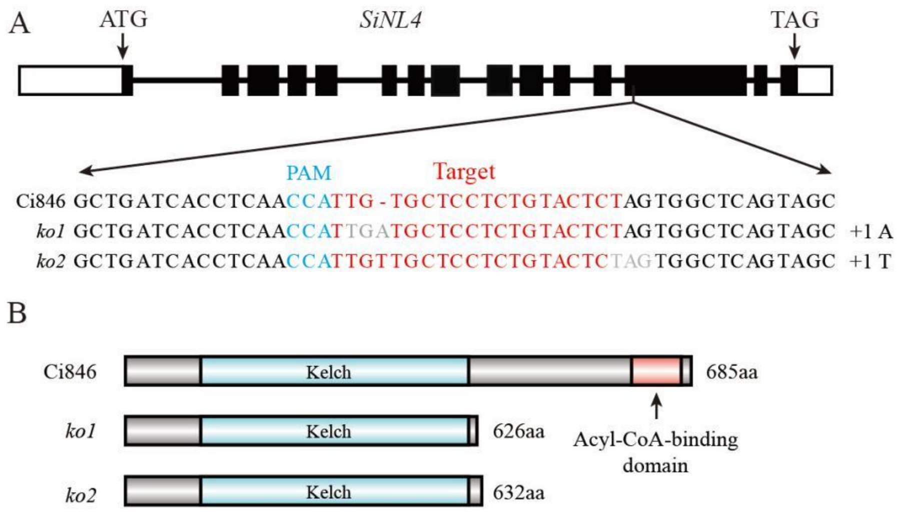

Figure S3 Sequence alignment of Ci846, *ko1*, and *ko2* and comparison of their protein structures. (A) Mutations at the target site of the *SiNL4* gene. (B) Truncated proteins produced by the SiNL4 mutations.

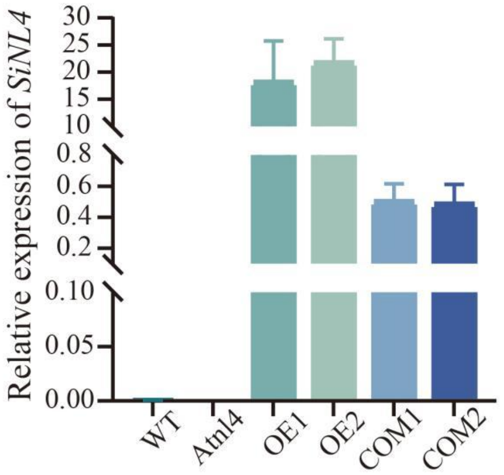

Figure S4 Relative expression levels of *SiNL4* in *Arabidopsis* Col-0, *Atnl4*, overexpression lines, and complementation lines.
